# Validating clinical feasibility of MRCAT and deep learning‐based synthetic CT images for cervical cancer patient

**DOI:** 10.1002/acm2.70332

**Published:** 2025-11-05

**Authors:** Dohyeon Yoo, Hojin Kim, Sangjoon Park, Hyeok Choi, Se Young Kim, Jin Sung Kim, Yong Bae Kim

**Affiliations:** ^1^ Department of Radiation Oncology Yonsei Cancer Center Yonsei University College of Medicine & Heavy Ion Therapy Research Institute Yonsei University College of Medicine Seoul Republic of Korea

**Keywords:** cervical cancer, deep‐learning, MR images, MRCAT, synthetic CT image

## Abstract

**Background:**

Various methods have been developed to generate synthetic computed tomography (CT) images from magnetic resonance (MR) images, including segmentation‐based approach with MR calculating attenuation (MRCAT) and deep learning (DL)‐based approach.

**Purpose:**

In this study, we aimed to validate the conventional radiotherapy (RT) planning process with MRCAT and DL‐based synthetic CT images for five patients with cervical cancer.

**Methods:**

DL‐based synthetic CT images of the five patients were inferred using a network trained with 40 pairs of CT and deformed, normalized T2‐weighted MR scans; MRCAT images were obtained from mDixon sequences for the tested cases only. On the synthetic CT images, the contouring process for organs‐at‐risk (OARs) was automatically performed with minor adjustments, while two experienced radiation oncologists defined target volumes. Simultaneous integrated boost plans (2.2/2.0/1.8 Gy with 25 fractions) were produced from a commercial treatment planning system (TPS) TomoTherapy.

**Results:**

The plans with two synthetic CT images were compared with those based on genuine CT images for the five test cases. High geometric similarity was confirmed for the planning target volume (PTV), with average dice similarity coefficient (DSC) of 0.844 for the DL‐based and 0.829 for the MRCAT images. The mean percentage difference in gross tumor volume (GTV) was 20.71 ± 34.28% for DL‐based synthetic CT and 30.31 ± 46.20% for MRCAT images. By contrast, PTV, encompassing GTV, exhibited minimal changes with an average increase of 0.37 ± 3.10% and 1.66 ± 7.62%, respectively. MRCAT images and DL‐based synthetic CT revealed significant differences, relative to true CT images, in the entire volume (*p = 0.03*) of the bladder and in V_20Gy_ and V_30Gy_ of the resultant plans for the bladder (*p = 0.029* and *0.063*), all plans generated on the synthetic CTs were clinically acceptable and met institutional for target coverage.

**Conclusion:**

MRCAT and DL‐based synthetic CT images demonstrated clinical applicability, achieving plan quality similar to that of plans based on genuine planning CT images.

## 1 INTRODUCTION

Radiation treatment planning, which aims to maximize the dose of radiation to the target volume while sparing the dose to organs‐at‐risk (OARs),^1^, ^2^, ^3^ should be performed based on patient‐specific computed tomography (CT) images. However, despite its widespread use and the benefits of CT images, CT‐based radiotherapy (RT) planning has some drawbacks, particularly regarding soft‐tissue contrast.^4^, ^5^ For example, RT planning for patients with cervical cancer necessitates accurate target volume delineation from the uterine body and surrounding structures of the bladder and rectum. The lack of soft tissue contrast in CT images can limit differentiation between normal tissue and target volume, potentially compromising the precision of the treatment plan.^6^, ^7^

By contrast, magnetic resonance (MR) images offer superior soft‐tissue contrast, enabling more accurate tumor delineation and potentially more precise radiation coverage.^8^, ^9^, ^10^ However, unlike CT images, MR images do not provide physical details such as electron density needed for dose calculation, making them supplementary in RT. To enhance utility of MR images for RT, generation of CT‐like images, known as synthetic CT images, from MR images has been actively investigated and developed for decades.^11^, ^12^, ^13^ Several approaches, such as atlas‐based,^14^, ^15^, ^16^ segmentation‐based,^17^, ^18^, ^19^ and learning‐based methods,^20^, ^21^, ^22^ have produced synthetic CT images from MR images for RT clinical applications.

Significant advancements have been made in achieving the goal of generating synthetic CT images from MR images, with considerable efforts dedicated to applying these innovations in actual RT planning. These endeavors focused on improving the potential of synthetic CT images and broadening the use of MR images to RT. While various methods have been developed, they generally fall into two categories relevant for clinical translation: commercially available, segmentation‐based systems integrated by MRI manufacturers (e.g., MRCAT), and rapidly advancing deep learning‐based models from the research community. A direct, head‐to‐head comparison evaluating the clinical feasibility of these two distinct paradigms for a specific treatment site like cervical cancer is essential for guiding clinical adoption, yet such validation studies remain limited. To fill this critical gap, this study aimed to validate the feasibility of two types of synthetic CT images from MRCAT and deep learning (DL)‐based approaches. We conducted the RT planning process from contouring to treatment planning and compared the resulting plans against those based on the genuine planning CT images.

## 2 MATERIALS AND METHODS

### 2.1 Patient cohort for DL

This study strictly adhered to applicable guidelines and regulations. The study protocol was approved by the Ethics Committee/Institutional Review Board of Severance Hospital, Yonsei University College of Medicine, Seoul, Republic of Korea (approval no. 4‐2022‐0311), which also determined that informed patient consent was not required for the retrospective analysis of patient images. Patient data were collected between December 2022 and April 2023. CT images were scanned using a Canon Aquilion LB CT simulator (Canon Medical Systems Corporation, Japan). By contrast, T2‐weighted MR imaging was performed using an MR Ingenia 3.0T simulator (Philips Healthcare, Amsterdam, Netherlands). The voxel spacing differed between the MR and CT images, being 1.06 × 1.06 × 3 mm^3^ for MR images and 0.76 × 0.76 × 3 mm^3^ for CT images. MR and CT scans were conducted on the same day, a few hours apart, to align the images from two modalities as closely as possible. Additionally, patients were advised to empty their bladders before undergoing MR and CT imaging to ensure consistency. DL‐based synthetic CT images for five patients with cervical cancer were acquired using a network trained with 40 pairs of CT and deformed, normalized T2‐weighted (T2W) MR images. MRCAT images for the same five cases were acquired using an mDixon sequence provided by the MR simulation (Ingenia 3.0T, Philips Healthcare).

### 2.2 Preparation of two types of synthetic CT images (DL‐based and MRCAT)

To generate DL‐based synthetic CT images from T2W MR images, we utilized a deep convolutional neural network (CNN), implemented in TensorFlow 1.14 and Python 3.6 on a personal workstation equipped with a Nvidia GTX Titan X GPU. The network was trained on pairs of T2W MR and CT images to replicate the anatomical accuracy and image quality of actual CT scans, with the objective of producing synthetic CT images that closely resemble real CT images. As previously mentioned, images were captured under empty bladder conditions on the same day, specifically with an empty bladder for consistency. To ensure a high degree of similarity between the image pairs for network training, several pre‐processing techniques were applied. First, to address intensity variations observed across different patients' MR images despite uniform scanning procedures, Nyul normalization was employed to standardize the intensity distribution.^23^ All images were then formatted into a 512 × 512 × 3 pseudo‐three‐dimensional (3D) matrix for training. Following this, a two‐step image registration process was performed to ensure precise spatial correspondence. The multi‐modal registration between the T2W MR and CT images for the training dataset was conducted using the precision treatment planning system (Accuray Incorporated, USA). This process involved an initial rigid registration to globally align the images, followed by deformable image registration (DIR) to account for local, nonrigid anatomical variations between the scans, such as changes in bladder and rectum filling. While quantitative registration error metrics were not calculated, the final alignment of each image pair was visually inspected by a clinical expert to ensure accurate spatial correspondence. This two‐step process is critical for deep learning as it ensures voxel‐to‐voxel anatomical consistency, a prerequisite for training the network to learn an accurate intensity mapping.

A detailed generative adversarial network (GAN) architecture, with a U‐Net‐based generator employing skip connections and a residual structure in the bottleneck for better gradient preservation, and a discriminator comprising five down‐sampling convolution blocks with ReLU activations and a final sigmoid function for binary classification. This architecture was used to generate the synthetic CT images. The adversarial training aimed to make the discriminator increasingly unable to distinguish between authentic and generated images, with 100 epochs and a batch size of 3 used as training parameters to achieve model convergence (Figure S1).^24^

The mDixon multi‐echo sequence was used as part of the commercial package for generating synthetic CT images, named MRCAT.^25^, ^26^, ^27^ This approach is characterized by using a single mDixon magnetic resonance imaging (MRI) sequence in conjunction with a proprietary algorithm to accurately simulate electron density information. The detailed workflow of the MRCAT synthetic CT image generation algorithm from mDixon in‐phase, water‐only, and fat‐only images are depicted in Figure S2. The dual‐echo 3D Cartesian mDixon acquisition integral to this method produces distinct image contrasts: fat‐only, water‐only, in‐phase, and out‐of‐phase sequences. By acquiring two echoes, this technique enables the derivation of water, fat, and in‐phase images from a singular acquisition, leveraging the frequency shift between fat and water protons. The initial image processing phase involves automatic body contouring and background removal, primarily utilizing the mDixon water and in‐phase images to delineate the body mask, with all external regions classified as air. Subsequently, a classification algorithm segregates the internal anatomy into bone and soft tissue compartments, with the latter further differentiated into adipose and muscle/water‐like tissues based on the in‐phase and fat images. Bone segmentation was refined with a pelvic bone model/atlas, distinguishing between cortical (compact) and spongy (trabecular) bone. This refinement was achieved by classifying voxels within the bone mask as compact bone if their in‐phase signal intensity was above a specific threshold, while the remainder were categorized as spongy bone. Following these segmentation steps, each voxel was assigned a bulk Hounsfield Unit value reflective of its classification, facilitated by the MRCAT algorithm's unique relative electron density calibration curve. This curve, which is slightly shallower than the one traditionally used at our institution for HU values exceeding 500, ensures the precise assignment of CT attenuation coefficients to the segmented tissues.

### 2.3 Target and organs at risk contouring technique

The contouring process on target volumes and OARs was almost identical to the procedure performed on the original planning CT images. OARs were automatically delineated using a commercial auto‐segmentation software (OncoSoft Coreline Soft Co., OncoSoft Inc.) with minor adjustments being performed later. For static organs with minimal expected variation, such as the femur heads, the contours from the original planning CT were first copied to the synthetic CT images and then manually refined by an oncologist if any adjustments were needed to fit the underlying anatomy. Two radiation oncologists (H. Choi and Y.B. Kim) delineated and conformed target volumes. For the gross tumor volume (GTV) delineation on the planning CT images, T2‐weighted MR images were rigidly registered and fused with the planning CT to provide a reference for tumor localization. However, the final contouring was performed based on the anatomical information and tumor extent visible on the planning CT images. Key organs included in the contouring process were the bladder, small bowel, femur heads (right and left), and rectum, highlighting the comprehensive scope of our anatomical assessment. Contouring was conducted separately for the original CT images and for the two synthetic CT images (DL‐based and MRCAT) to allow a detailed comparison of contouring consistency and accuracy across different imaging modalities.

### 2.4 Dosimetric evaluation of two different types of synthetic CT images

To evaluate the clinical feasibility of DL‐based synthetic CT and MRCAT images, treatment was planned for five patients with cervical cancer using the Accuray Precision treatment planning system (Accuray Incorporated, Sunnyvale, CA) Version 2.0.1.1. Simultaneous integrated boost (SIB) plans (2.2/2.0/1.8 Gy with 25 fractions) were generated. Plan constraints, summarized in Table 1, follow our institutional protocol. For the planning target volume (PTV), a minimum of 95% coverage with the prescribed dose was aimed for, allowing a variation down to 90%. The maximum dose (D_0.03cc_) should not exceed 107% of the prescribed dose, with an acceptable variation up to 110%. OARs such as the rectum, bladder, bowel, and femur heads had defined constraints to balance efficacy and safety. Based on these constraints, plans with synthetic CT images were compared with original CT plans for the five cases.

**TABLE 1:** Plan constraints for OARs and PTV.

Name of structure	Dosimetric parameter	Per protocol	Variation acceptable
PTV	D_95%_ (% of PDD)	≥ 95%	≥ 90%
	D_0.03cc_ (% of PDD)	≤ 107%	≤ 110%
Rectum	D_50%_ (Gy)	≤ 45	≤ 54
	D_0.03cc_ (Gy)	≤ 50	≤ 55
Bladder	D_50%_ (Gy)	≤ 45	≤ 55
	D_0.03cc_ (Gy)	≤ 50	≤ 57.5
Bowel	D_30%_ (Gy)	≤ 40	≤ 50
	D_0.03cc_ (Gy)	≤ 54	≤ 61
Femoral Heads	V_45Gy_ (cc)	≤ 200 cc	≤ 250 cc
	D_0.03cc_ (Gy)	≤ 50	≤ 55

### 2.5 Evaluation and statistical analysis

Clinical feasibility of the two synthetic CT images was assessed in two phases—volume of structures and dose distribution of the resulting plan. The assessment considered GTV, PTV, and OARs, including the bowel, bladder, rectum, and femur heads (right and left). For all categories, a comprehensive statistical analysis was conducted to evaluate the differences between the synthetic CT images (both DL‐based and MRCAT) and the original CT images. A paired t‐test was applied for each comparison to determine if there were statistically significant differences in the volumes and dosimetric indices across imaging modalities. This analysis, performed using IBM SPSS Statistics version 26, involved calculating *p*‐values to assess the likelihood that any observed differences occurred by chance, with statistical significance set at *p *< 0.05. By using paired t‐tests, we ensured that each patient served as their own control across different image sets, enhancing the reliability of the findings and minimizing variability due to individual anatomical differences. First, GTV, PTV, and the volume of OARs were compared to evaluate the precision of organ delineation and volume estimation between original and two types of synthetic CT images. The volumetric analysis helped validate the clinical relevance of synthetic CT images in replicating anatomical details necessary for effective treatment planning.

A quantitative analysis for the resulting dose distributions was conducted using conformity index (CI), dose homogeneity index (DHI), homogeneity index (HI), maximum dose (GTV_Dmax_ and PTV_Dmax_), and percentage of volume receiving 95% of the prescribed dose (GTV_V95%_ and PTV_V95%_). The indices were defined as follows:

(1) $$CI=V_{RI}/TV\;$$

(2) $$DHI=D_{\geq 95\%\;\left(withinPTV\right)}/D_{\geq 5\%\;\left(withinPTV\right)}\;$$

(3) $$HI=I_{max}/RI\;$$

CI in Equation (1) indicated how well the irradiated volume conforms to the target volume, where $V_{RI}$ is the volume receiving the reference isodose, and TV is the target volume. DHI in Equation (2) assessed the uniformity of dose distribution within the PTV, where $D_{\geq 95\%}$ is the dose covering at least 95% of the PTV, and $D_{\geq 5\%}$ is the dose covering at least 5% of the PTV. HI in Equation (3) provided a measure of dose homogeneity across the target volume, where $I_{max}$ is the maximum isodose within the target, and RI is the reference isodose. For the OARs, the mean dose (D_mean_), and percentage volume receiving 5, 10, 20, and 30 Gy (V_5Gy_, V_10Gy_, V_20Gy_, and V_30Gy_) were evaluated alongside the volume. Finally, the evaluation involved various metrics, including dose–volume histograms (DVHs) for GTV, PTV, and OAR mean dose comparisons, and assessments of conformity and homogeneity indices.

Additionally, the dice similarity coefficient (DSC) was calculated to compare the spatial similarity of contours for target volumes and OARs between the synthetic and original CT images (MATLAB R2024a). The DSC was defined as follows:

$$DSC=\frac{2\left|A\right.\cap \left.B\right|}{\left|A\right|+\left|B\right|}$$

where A and B represent the voxel sets of the compared contours. A DSC value ranges from 0 (no overlap) to 1 (perfect overlap). This metric provides a more comprehensive assessment than volumetric comparison alone, as it directly quantifies the degree of spatial alignment between contours and thus avoids potentially misleading conclusions.

## 3 RESULTS

**FIGURE 1:**
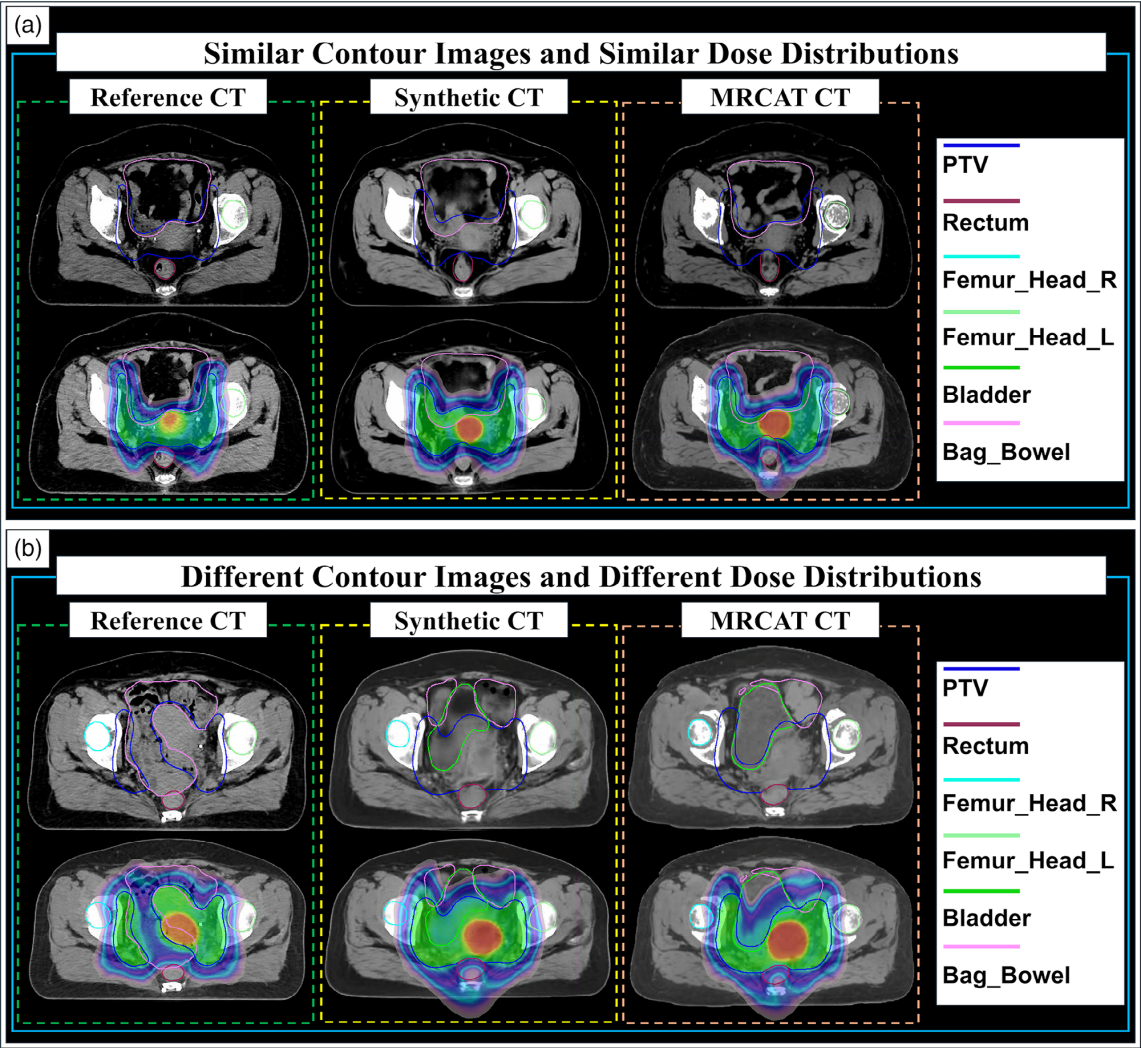
Comparison of contour images and dose distribution maps for the same treatment plan. (a) Similar contour images and dose distribution maps for the same computed tomography (CT) slice number for patient 3; (b) Different contour images and dose distribution maps for the same CT slice number for patient 1.

Organ‐specific contour delineation results alongside the calculated dose distribution outcomes are presented in Figure 1. This figure includes the target volumes that the experienced physician delineated (blue lines for GTV, green lines for the bladder, and pink lines for the small bowel). From left to right, the images represent the original CT, DL‐based synthetic CT, and MRCAT synthetic CT images, respectively. The synthetic CT images were derived from the MR images, which were slightly different from the planning CT images in the anatomical details despite minimizing the time interval between MR and CT scans. Figure 1a illustrates cases where the contour delineations and dose distributions closely resemble those from the original CT images. Contrarily, Figure 1b reveals that the contours delineated on the synthetic CT images differed from those on the genuine planning CT images, thus yielding different dose distributions.

Numerical information regarding volume of GTV and PTV delineated on two types of synthetic CT images, with reference to that on the original planning CT images are listed in Table 2. The mean GTV volume was 34.41 ± 6.49 cc for DL‐based synthetic CT and 36.99 ± 10.18 cc for MRCAT, though these differences were not statistically significant (*p >* *0.05*) compared to the original CT value of 29.70 ± 6.73 cc. By contrast, the mean PTV volume showed minimal changes, with values of 769.86 ± 78.05 cc for DL‐based synthetic CT and 776.47 ± 53.68 cc for MRCAT, compared to 769.00 ± 93.37 cc for the original CT. Specifically, the patient‐specific variations of GTV volume delineated on the DL‐based synthetic CT images ranged from −21.13% to 59.76%, and the variations on MRCAT were between −22.76% and 88.12%, compared with the GTVs on the original CT. The assessment of PTV revealed a smaller range of variations, with DL‐based synthetic CT and MRCAT volumes exhibiting a maximum increase of 4.20% and 9.48%, respectively, and a decrease by up to −8.70% for MRCAT synthetic CT.

**TABLE 2:** Comparative volumetric analysis of target volumes (GTV and PTV) and bladder. Individual patient data, percentage differences, group summaries (Mean ± SD), and *p*‐values are presented.

	GTV_volume_ (cc)				
	Original CT	DL‐based synthetic CT	MRCAT	% Difference with	
				DL‐based synthetic CT	MRCAT
Patient 1	40.33	31.81	37.54	−21.13%	−6.92%
Patient 2	22.22	30.28	37.62	36.27%	69.31%
Patient 3	27.78	44.38	52.26	59.76%	88.12%
Patient 4	27.10	37.26	33.55	37.49%	23.80%
Patient 5	31.07	28.33	24.00	−8.82%	−22.76%
Mean ± SD (*p‐value*)	29.70 ± 6.73 (‐)	34.41 ± 6.49 (*0.400*)	36.99 ± 10.18 (*0.254*)	20.71 ± 34.28 (‐)	30.31 ± 46.20 (‐)

	PTV_volume_ (cc)				
	Original CT	DL‐based synthetic CT	MRCAT	% Difference with	
				DL‐based synthetic CT	MRCAT
Patient 1	651.11	668.13	708.39	2.61%	8.80%
Patient 2	889.98	863.61	811.95	−2.89%	−8.70%
Patient 3	826.27	825.34	846.83	−0.11%	2.49%
Patient 4	704.97	734.61	771.82	4.20%	9.48%
Patient 5	772.68	757.63	743.37	−1.95%	−3.79%
Mean ± SD (*p‐value*)	769.00 ± 93.37 (‐)	769.86 ± 78.05 (*0.975*)	776.47 ± 53.68 (*0.784*)	0.37 ± 3.10 (‐)	1.66 ± 7.62 (‐)

	Bladder_volume_ (cc)				
	Original CT	DL‐based synthetic CT	MRCAT	% Difference with	
				DL‐based synthetic CT	MRCAT
Patient 1	56.08	163.03	196.5	190.71%	250.48%
Patient 2	59.61	58.79	88.9	−1.38%	49.14%
Patient 3	69.25	105.28	135.42	52.03%	95.14%
Patient 4	94.92	198.74	220.19	109.38%	131.97%
Patient 5	106.75	134.78	134.78	26.26%	26.26%
Mean ± SD (*p‐value*)	77.32 ± 22.40 (‐)	132.12 ± 53.75 (*0.054*)	155.16 ± 51.04 (*0.030*)	75.40 ± 76.77 (‐)	110.60 ± 88.58 (‐)

The differences in volume between planning and synthetic CT images were larger in OARs. The differences were particularly notable in the bladder, in which DL‐based synthetic CT images revealed an increase of up to 190.71% and MRCAT up to 250.48% in patient 1. For the small bowel, the DL‐based synthetic CT images had a significant decrease by −56.56% and an increase by 76.45% for patients 1 and 5, respectively, whereas MRCAT led to an increase by 76.45% in patient 5. Differences in the rectum volume between the planning and synthetic CT images were also significant. The rectum volumes delineated on the DL‐based and MRCAT synthetic images were approximately twice as large as those on the planning CT images, and the MRCAT synthetic images yielded larger differences from the reference in patients 2 and 5 than the DL‐based synthetic images. For the femur heads, the DL‐based and MRCAT synthetic images led to similar, consistent contouring results, in which the differences from the reference ranged from ‐25.38% to 13.12% for DL‐based synthetic CT, and from −24.26% to 12.12% for MRCAT synthetic CT (detailed OAR volume difference values are provided in Table S1). To investigate the underlying cause of the dosimetric differences, the Hounsfield Unit (HU) values were quantified. The detailed analysis, presented in Table S2, revealed a systematic underestimation of HU values by both synthetic methods, particularly for bony structures and the bladder. Notably, the DL‐based approach consistently demonstrated a smaller mean deviation from the original CT values, suggesting higher overall HU accuracy compared to the MRCAT method.

**FIGURE 2:**
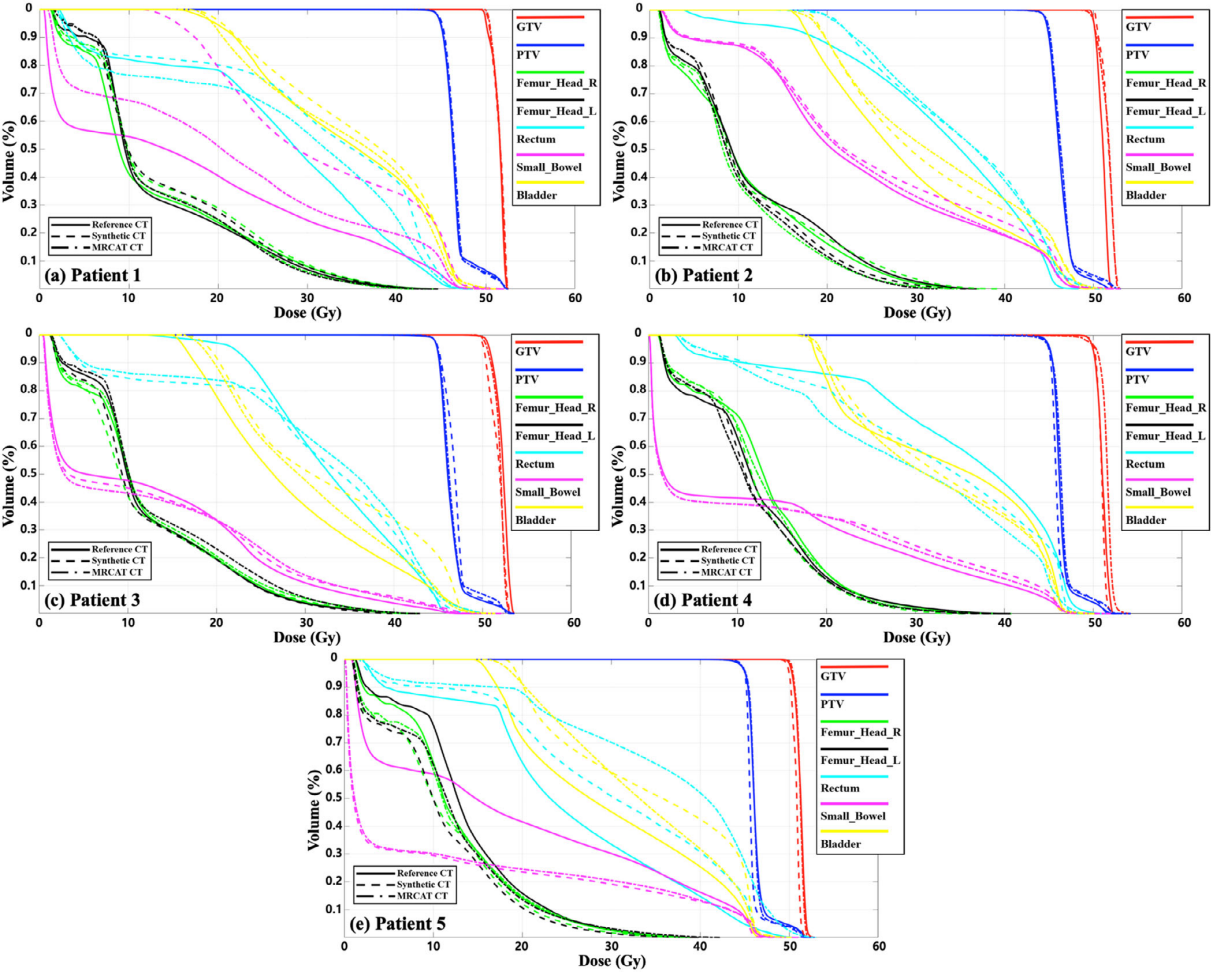
Dose–volume histogram (DVHs) calculated from original computed tomography (CT) images, deep learning (DL)‐based synthetic CT, and MR for calculating attenuation (MRCAT) CT images for a cohort of five patients with cervical cancer (patient 1 to patient 5, labeled as a to e). (The dashed line represents the original CT, the dotted lines indicate DL‐based synthetic CT, and the dashed dotted line denotes MRCAT).

Treatment planning was performed on the planning and two synthetic CT images with the contours delineated in the target volumes and OARs, which yielded DVHs (Figure 2). When comparing three plans (based on planning CT, DL‐based synthetic CT, and MRCAT images), the DVHs on GTV, PTV and the femur heads behaved similarly for the five tested cases, whereas the variations on DVHs occurred in the bladder, small bowel and rectum. The degree of these observed variations may be proportional to the discrepancy in the delineated volumes between the planning and synthetic CT images.

A detailed quantitative comparison of key dosimetric indices for the target volumes and OARs is presented in the supplementary materials. Table S3 provides a comprehensive comparison of the dose distributions for the planning CT and the two synthetic CT images. Furthermore, Table S4 contains a detailed statistical analysis for the OARs, including p‐values and 95% confidence intervals (CIs) for the dosimetric comparison. These tables offer a complete and granular assessment of the dosimetric performance for each synthetic CT method.

Results of comparing DSC values between the original CT and synthetic CTs are presented in Table 3. Results of comparing DSC values between two different types of synthetic CTs are presented in Table 4. DSC values between the original and synthetic CT images reveal different patterns of similarity for different targets and OARs.

**TABLE 3:** Dice similarity coefficient (DSC) values between original CT vs. synthetic CTs (DL‐based and MRCAT) for target and OARs. Values are presents as mean ± SD, with *p*‐values from paired *t*‐tests comparing two methods.

Structure	Type of synthetic CT	Patient 1	Patient 2	Patient 3	Patient 4	Patient 5	Mean ± SD
GTV	DL‐based Synthetic CT	0.5714	0.6045	0.6473	0.7719	0.7559	0.670 ± 0.090
	MRCAT Synthetic CT	0.6105	0.3966	0.5997	0.8484	0.7747	0.646 ± 0.174
	*p‐value*						*0.814*
PTV	DL‐based Synthetic CT	0.7874	0.8534	0.8805	0.8399	0.8608	0.844 ± 0.035
	MRCAT Synthetic CT	0.7752	0.7857	0.8761	0.8594	0.8483	0.829 ± 0.046
	*p‐value*						*0.463*
Rectum	DL‐based Synthetic CT	0.7220	0.5284	0.6242	0.8151	0.7085	0.680 ± 0.111
	MRCAT Synthetic CT	0.8252	0.4685	0.6002	0.8218	0.6528	0.674 ± 0.147
	*p‐value*						*0.953*
Bladder	DL‐based Synthetic CT	0.5737	0.7241	0.7152	0.6619	0.7781	0.691 ± 0.076
	MRCAT Synthetic CT	0.5536	0.5271	0.6468	0.6693	0.7475	0.629 ± 0.077
	*p‐value*						*0.235*
Femur_Head (Right)	DL‐based Synthetic CT	0.8640	1	0.9525	0.8943	0.9135	0.925 ± 0.056
	MRCAT Synthetic CT	0.8552	1	0.9882	0.9017	0.9135	0.932 ± 0.059
	*p‐value*						*0.787*
Femur_Head (Left)	DL‐based Synthetic CT	0.8525	1	0.9762	0.8765	0.9215	0.925 ± 0.059
	MRCAT Synthetic CT	0.8613	1	0.9983	0.8794	0.9215	0.932 ± 0.057
	*p‐value*						*0.771*
Small_Bowel	DL‐based Synthetic CT	0.5732	0.9366	0.9664	0.9686	0.5743	0.804 ± 0.199
	MRCAT Synthetic CT	0.7695	0.9107	0.9710	0.9667	0.5743	0.838 ± 0.160
	*p‐value*						*0.710*

**TABLE 4:** Dice similarity coefficient (DSC) values between two types of synthetic CTs (DL‐based versus MRCAT) for target and OARs.

GTV	DL‐based vs. MRCAT	PTV	DL‐based vs. MRCAT
Patient 1	0.8355	Patient 1	0.8927
Patient 2	0.6375	Patient 2	0.8664
Patient 3	0.8583	Patient 3	0.8894
Patient 4	0.8652	Patient 4	0.8796
Patient 5	0.7557	Patient 5	0.8834
Mean ± SD	0.7904 ± 0.0959	Mean ± SD	0.8823 ± 0.0102
Rectum	DL‐based vs. MRCAT	Bladder	DL‐based vs. MRCAT
Patient 1	0.7743	Patient 1	0.8343
Patient 2	0.7606	Patient 2	0.6974
Patient 3	0.8597	Patient 3	0.8636
Patient 4	0.8679	Patient 4	0.8996
Patient 5	0.7696	Patient 5	0.8844
Mean ± SD	0.8064 ± 0.0527	Mean ± SD	0.8359 ± 0.0812
Femur_Head_R	DL‐based vs. MRCAT	Femur_Head_L	DL‐based vs. MRCAT
Patient 1	0.9422	Patient 1	0.9376
Patient 2	1	Patient 2	1
Patient 3	0.9641	Patient 3	0.9777
Patient 4	0.9569	Patient 4	0.9546
Patient 5	1	Patient 5	1
Mean ± SD	0.9726 ± 0.0262	Mean ± SD	0.9740 ± 0.0277
Small_Bowel	DL‐based vs. MRCAT		
Patient 1	0.7130		
Patient 2	0.9246		
Patient 3	0.9813		
Patient 4	0.9975		
Patient 5	1		
Mean ± SD	0.9233 ± 0.1214		

For the GTV, the mean DSC values were 0.670 ± 0.090 for DL‐based synthetic CT and 0.646 ± 0.174 for MRCAT images, with no significant difference between the two methods (*p = 0.814*). By contrast, the PTV showed a higher degree of similarity, with mean DSC values of 0.844 ± 0.035 for DL‐based synthetic CT and 0.829 ± 0.046 for MRCAT images, indicating that synthetic CTs may be more effective in accurately reproducing the PTV, which is crucial for treatment planning.

For OARs that experience volume changes over time, such as the rectum and bladder, the average DSC values were 0.680 and 0.691, respectively, for DL‐based CT. For MRCAT synthetic CT, the average DSC values were slightly lower, at 0.674 for the rectum and 0.629 for the bladder. Moreover, relatively stable organs such as the femur heads and small bowel have higher DSC values. The femur heads have average DSC values of 0.925 (right) and 0.925 (left) for DL‐based CT, indicating excellent similarity. The small bowel reveals an average DSC of 0.804 in DL‐based CT. These results highlight the reliability of synthetic CTs in reproducing the anatomy of more static structures.

The two types of synthetic CTs for different targets and OARs reveal a high degree of similarity, with some variations. For the GTV, the average DSC is 0.7904, indicating good agreement between DL‐based and MRCAT synthetic CTs. The PTV exhibited a similar trend, with an average DSC of 0.8823, suggesting that both synthetic CTs were highly consistent in replicating the PTV. Dynamic organs such as the rectum and bladder have average DSC values of 0.8064 and 0.8359, respectively, indicating that although replicating these organs has challenges, the synthetic CTs achieved substantial anatomical accuracy. Stable organs, such as the femur heads, have very high DSC values, with averages of 0.9726 (right) and 0.9740 (left). The small bowel also has a high average DSC value of 0.9233. These results reflect a consistent trend where stable structures are reproduced with greater accuracy using synthetic CTs.

## 4 DISCUSSION

Cervical cancer is highly radiosensitive, making radiation therapy (RT) a crucial treatment method. Prognosis typically depends on the patient's response to RT within 2 months, with quicker complete remission indicating a better outcome. Radiation oncologists must deliver an adequate dose while minimizing side effects. The introduction of intensity‐modulated RT (IMRT) highlighted the importance of precisely defining the target and OARs. MR simulation enhances target and OAR definition, although it requires merging CT and MR images for accurate treatment planning. Synthetic CTs based on MR simulation could reduce the need for separate scans, minimizing inconvenience and cost. Nowadays, MRI manufacturers have developed synthetic CT generating software, using segmentation‐based approaches. These were approved by the FDA in the US, suggesting that synthetic CT generated from MR simulators could be applied in clinical radiotherapy.

The study determined minimal variation in the PTV across CT images, which may possibly be attributable to PTV definition based on pelvic vessels, similar to bony landmarks in 3D‐conformal RT. However, substantial differences in the GTV were observed. This study utilized the SIB technique, which increased the fractional dose to the GTV, possibly causing these differences. Adaptive CT during RT and MR LINAC allow for replanning based on onboard MR images, addressing GTV uncertainty owing to tumor regression. Vaginal immobilization devices can also limit uterine movement.^28^ Dosimetric differences were noted in dose distributions for the rectum, small bowel, and bladder, attributed to the time gap between CT and MR imaging sessions. Internal content variability likely contributed to contour differences and DVH discrepancies.

These discrepancies in contouring and dosimetry are consistent with previous studies on synthetic CT, where differences in OAR volumes, particularly for the bladder and rectum, have been observed due to their dynamic nature and variability between imaging sessions. It was also found that both DL‐based and segmentation‐based synthetic CT images are vulnerable to elucidating gas or air cavity and soft tissue variations adjacent to the cervix.^29^, ^30^ It may require a more minute discretization in the segmentation‐based approach, and more advanced architecture and well‐aligned datasets for the DL‐based approaches. Nevertheless, DL‐based and segmentation‐based synthetic CT generations are considered more robust than the previous atlas‐based approach as these are not dependent on the atlas library and the deformation accuracy between the MR and CT images.^31^, ^32^ The significant volumetric discrepancies observed, particularly in the bladder and GTV, warrant further discussion regarding their clinical impact. While attributed to physiological variability, these findings highlight a critical challenge in conventional radiotherapy, where a single plan is often delivered over multiple fractions. Such large inter‐scan variations could compromise treatment accuracy, potentially leading to underdosing of the target volume or excessive irradiation of adjacent OARs. This underscores the importance of rigorous patient preparation protocols and highlights the value of superior soft‐tissue contrast provided by MR imaging for more accurate delineation, even if performed only at the simulation stage. Our results suggest that while synthetic CTs are feasible for planning, the underlying anatomical variability they reveal necessitates careful consideration of planning margins and robust quality assurance to ensure treatment fidelity. The primary contribution of this study is the rigorous head‐to‐head validation of two distinct and clinically crucial paradigms—a commercially available system (MRCAT) and a state‐of‐the‐art deep learning model—for cervical cancer radiotherapy.

This direct comparison advances the field by providing clinicians with critical evidence on the practical strengths and limitations of each approach, addressing a notable gap in the literature where such direct comparative data is scarce. By assessing the entire clinical workflow from contouring to final dose distribution, our work provides a practical benchmark that can guide clinical adoption and future research in MR‐only radiotherapy.

Differences in OARs, particularly the bladder, rectum, and small bowel, were expected owing to their mobility and volume sensitivity. Despite efforts to minimize bladder volume differences, tumor‐induced pressure often prevents complete emptying. The rectum's appearance varies with content, and although enemas can be administered before imaging, they are impractical before each treatment session. Small bowel movement also introduces variability. To mitigate these variations, the most important point is to make MR simulation as similar as possible to the actual treatment setup. For example, from our observation, the coil for MR images should be positioned slightly apart from the patient to avoid compressing the body. Nevertheless, treatment plans on synthetic CT images met dose constraints and achieved similar quality to planning CT images.

This conclusion of similar quality, despite the noted statistical differences in GTV and certain OAR dosimetric, is based on two key factors. First, while GTV delineation varied significantly due to superior soft‐tissue contrast on the MR‐based images, the treatment plans were optimized using the planning target volume (PTV), which showed minimal volumetric changes across all image sets (average increase of 0.37% and 1.66% for DL‐based and MRCAT, respectively). Second, and most importantly, our definition of ‘similar plan quality’ refers to the fact that all plans successfully met predefined clinical goals for target coverage and OAR sparing as outlined in our institutional protocol. Therefore, while some dosimetric parameters for mobile organs like the bladder were statistically different, these variations did not compromise the clinical acceptability of the final treatment plans. This physiological variability was also reflected in the spatial overlap of the contours, as measured by the DSC. DSC values for stable structures, such as the PTV and the femur heads, exhibited high similarity between DL‐based CT, MRCAT images, and the original CT, whereas variable organs such as the rectum and bladder showed greater DSC variability.

A major limitation of this study is the small sample size of five patients, which significantly restricts the generalizability of the findings. Therefore, the results presented should be considered preliminary. While this study serves as an important proof‐of‐concept, future large‐scale studies are essential to validate our conclusions. To ensure robustness and readiness for broad clinical adoption, such follow‐up studies should ideally involve expanded patient cohorts from multiple institutions and validation on external datasets. Furthermore, while all image registrations were visually verified for clinical accuracy, a quantitative assessment of the registration error (e.g., using TRE or landmark analysis) was not performed, which constitutes another limitation of this study. The current work utilized the convolutional neural network‐based approach, while many state‐of‐the‐art generative networks have been proposed. Additionally, the study focused on synthetic CT images from a DL GAN‐based network and a segmentation‐based approach with MRCAT. Future research should explore advanced generative networks such as vision transformers and diffusion models, which may improve synthetic CT translation accuracy. For instance, vision transformers, with their global self‐attention mechanisms, could better capture long‐range spatial relationships across the entire pelvic anatomy. This may help address the challenge of organ variability (e.g., bladder and rectum filling) by creating more anatomically consistent synthetic CTs. Furthermore, diffusion Models, which generate images through a probabilistic reverse process, could offer a path to more realistic and accurate (HU estimation. By learning the underlying data distribution, they might reduce the HU estimation errors observed in both current DL and segmentation‐based methods, particularly for challenging tissues like bone and air pockets. Exploring these state‐of‐the‐art models could significantly improve the robustness and fidelity of synthetic CT generation.

## 5 CONCLUSIONS

This study evaluated the clinical feasibility of MRCAT and DL‐based synthetic CT images generated from T2W MR images for RT planning for cervical cancer. Notable discrepancies in the bladder, rectum, and small bowel underscored the challenges posed by physiological variations and the necessity for meticulous contouring. These observations emphasized the critical need for developing and adhering to standardized imaging protocols for MR and CT imaging sessions, aiming to bolster the precision and clinical utility of synthetic CT technologies for RT planning. Nevertheless, our findings revealed that both synthetic CT approaches could closely emulate the dosimetric characteristics of traditional CT scans, ensuring uniform dose distributions across both target volumes and critical anatomical structures. In addition to highlighting the potential of synthetic CT, this investigation confirmed its applicability in clinical settings.

## AUTHOR CONTRIBUTIONS

Dohyeon Yoo, Hojin Kim, Sangjoon Park, Hyeok Choi, Se Young Kim, Jin Sung Kim, and Yong Bae Kim contributed to the conception and design of the study. Data were collected by Dohyeon Yoo, Hyeok Choi, and Se Young Kim. Statistical analysis was performed by Dohyeon Yoo. Dohyeon Yoo and Hojin Kim drafted and revised the manuscript. Hojin Kim, Sangjoon Park, and Yong Bae Kim confirm the authenticity of all the war data. All the authors read and approved the final version of the manuscript.

## CONFLICT OF INTEREST STATEMENT

The authors declare no conflict of interest.

## ETHICS STATEMENT

This study was approved by the Institutional Review Board of Severance Hospital (approval no. 4‐2022‐0311), and the requirement for informed consent was waived because of the retrospective study design.

## Supporting information



Supporting Information

## Data Availability

The datasets generated during the current study will be available from corresponding author on reasonable request.
