# Supplementary material for: Validating clinical feasibility of MRCAT and deep learning‐based synthetic CT images for cervical cancer patient
Source: J Appl Clin Med Phys. 2025 Nov 5;26(11):e70332. doi: 10.1002/acm2.70332 (PMC12589824; doi:10.1002/acm2.70332)
Supplement: Supplementary file 1 — Supporting Information [file ACM2-26-e70332-s001.docx]

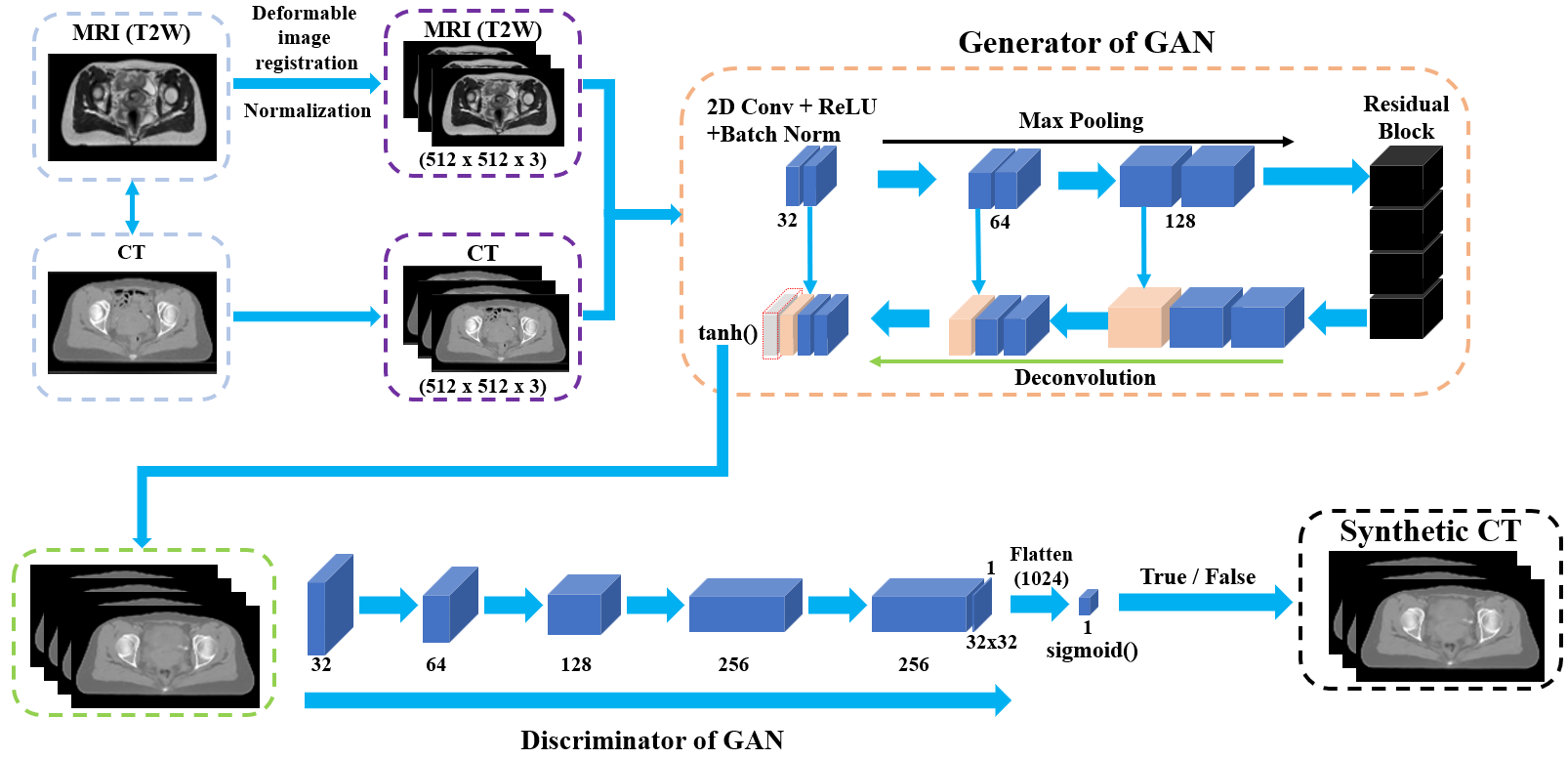


Figure S1. Specific workflow of the pre-processing and network training steps for generating DL-based synthetic CT.


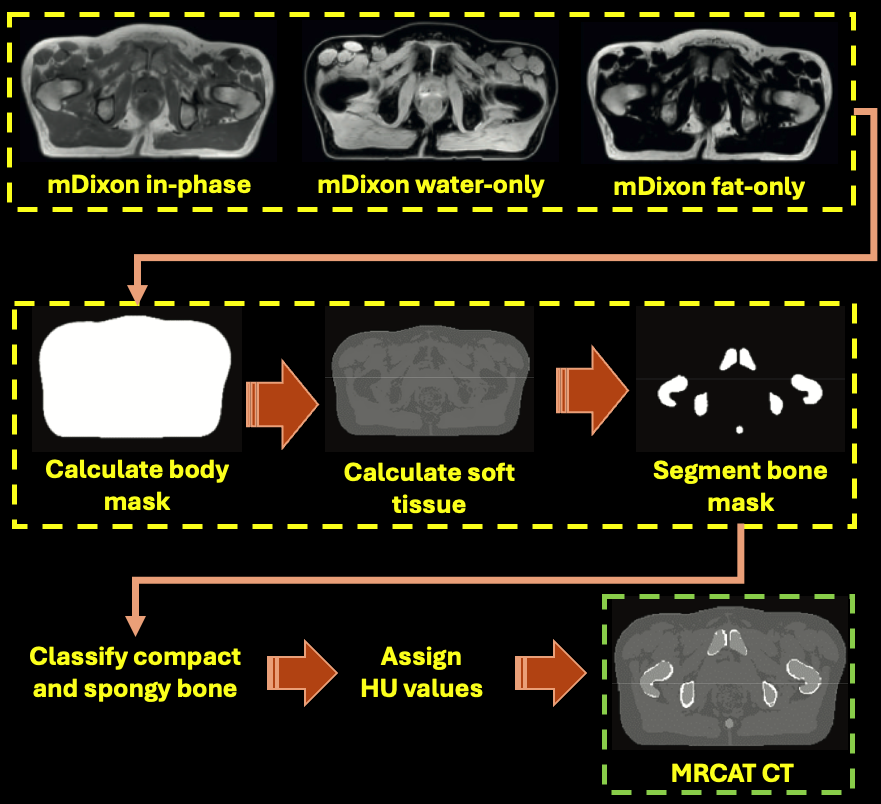


Figure S2. MRCAT synthetic CT generation algorithm from mDixon in-phase, water-only, and fat-only MR images.

Table S1. Comparative volumetric analysis of OARs in two different types of synthetic CT based on the original CT images.

|  | **Bladder_volume_ (cc)** | | | | |
| --- | --- | --- | --- | --- | --- |
|  | **Original** | **DL-based Synthetic CT** | **MRCAT** | **% Difference with** | |
|  |  |  |  | **Synthetic CT** | **MRCAT** |
| **Patient 1** | 56.08 | 163.03 | 196.5 | 190.71% | 250.48% |
| **Patient 2** | 59.61 | 58.79 | 88.9 | -1.38% | 49.14% |
| **Patient 3** | 69.25 | 105.28 | 135.42 | 52.03% | 95.14% |
| **Patient 4** | 94.92 | 198.74 | 220.19 | 109.38% | 131.97% |
| **Patient 5** | 106.75 | 134.78 | 134.78 | 26.26% | 26.26% |
| **Average % Difference** | | | | 75.40% | 110.60% |
|  | **Small_Bowel_volume_ (cc)** | | | | |
|  | **Original** | **DL-based Synthetic CT** | **MRCAT** | **% Difference with** | |
|  |  |  |  | **Synthetic CT** | **MRCAT** |
| **Patient 1** | 2299.98 | 999.16 | 1511.39 | -56.56% | -34.29% |
| **Patient 2** | 1341.21 | 1201.64 | 1142.97 | -10.41% | -14.78% |
| **Patient 3** | 3163.25 | 3165.8 | 3137.93 | 0.08% | -0.80% |
| **Patient 4** | 1817.87 | 1736.61 | 1734.60 | -4.47% | -4.58% |
| **Patient 5** | 886.28 | 1563.88 | 1563.88 | 76.45% | 77.02% |
| **Average % Difference** | | | | 1.02% | 4.51% |
|  | **Rectum_volume_ (cc)** | | | | |
|  | **Original** | **DL-based Synthetic CT** | **MRCAT** | **% Difference with** | |
|  |  |  |  | **Synthetic CT** | **MRCAT** |
| **Patient 1** | 66.52 | 86.53 | 59.60 | 32.07% | -9.04% |
| **Patient 2** | 62.27 | 80.93 | 108.65 | 29.97% | 74.48% |
| **Patient 3** | 35.42 | 70.48 | 76.01 | 98.98% | 114.60% |
| **Patient 4** | 73.69 | 72.90 | 76.73 | -1.07% | 4.13% |
| **Patient 5** | 94.89 | 126.43 | 145.96 | 32.24% | 53.82% |
| Average % Difference | | | | 38.44% | 47.60% |
|  | **Femur_Head_R_volume_ (cc)** | | | | |
|  | **Original** | **DL-based Synthetic CT** | **MRCAT** | **% Difference with** | |
|  |  |  |  | **Synthetic CT** | **MRCAT** |
| **Patient 1** | 106.48 | 82.96 | 81.31 | -22.09% | -23.64% |
| **Patient 2** | 127.22 | 127.22 | 127.22 | 0.00% | 0.00% |
| **Patient 3** | 134.68 | 125.61 | 132.29 | -6.73% | -1.77% |
| **Patient 4** | 133.19 | 108.64 | 109.70 | -18.43% | -17.64% |
| **Patient 5** | 87.38 | 98.72 | 98.72 | 12.98% | 12.98% |
| **Average % Difference** | | | | -6.85% | -6.01% |
|  | **Femur_Head_L_volume_ (cc)** | | | | |
|  | **Original** | **DL-based Synthetic CT** | **MRCAT** | **% Difference with** | |
|  |  |  |  | **Synthetic CT** | **MRCAT** |
| **Patient 1** | 102.54 | 76.52 | 77.66 | -25.38% | -24.26% |
| **Patient 2** | 118.25 | 118.25 | 118.25 | 0.00% | 0.00% |
| **Patient 3** | 137.53 | 136.90 | 137.53 | -0.46% | 0.00% |
| **Patient 4** | 124.57 | 102.30 | 105.60 | -17.88% | -15.23% |
| **Patient 5** | 83.32 | 94.25 | 94.25 | 13.12% | 12.12% |
| **Average % Difference** | | | | -6.12% | -5.47% |

Table S2. Mean difference of Hounsfield Unit (HU) values ($\pm$ standard deviation) between synthetic CTs and original Ct across five patients.

| **Structure** | **Mean Difference** $\boldsymbol{\pm}$ **Standard Deviation** | |
| --- | --- | --- |
|  | **Original CT vs.**  **DL-based synthetic CT** | **Original CT vs.**  **MRCAT CT** |
| **PTV** | -10.40 $\pm$ 15.88 HU | -21.64 $\pm$ 9.01 HU |
| **Rectum** | -10.39 $\pm$ 32.12 HU | -11.00 $\pm$ 17.62 HU |
| **Bladder** | -127.36 $\pm$ 69.80 HU | -292.00 $\pm$ 72.92 HU |
| **Femur_Head_R** | -7.10 $\pm$ 31.23 HU | -53.93 $\pm$ 26.86 HU |
| **Femur_Head_L** | -13.85 $\pm$ 50.19 HU | -67.76 $\pm$ 35.62 HU |
| **Small_Bowel** | -14.71 $\pm$ 60.61 HU | -8.74 $\pm$ 61.79 HU |

Table S3. Dosimetric comparison of DL-based synthetic CT and MRCAT against ground truth for target volume (GTV and PTV), including p-values with 95% confidence interval (CI) (below each result in DL-based and MRCAT synthetic CT).

| **GTV** | **Ground truth (original CT)** | **DL-based synthetic CT** | **MRCAT** |
| --- | --- | --- | --- |
| **CI** | 27.342 | 25.536  (0.310)  (-0.277, 7.889) | 23.062  (0.390)  (-1.348, 9.908) |
| **DHI** | 0.962 | 0.963  (0.840)  (-0.005, 0.004) | 0.962  (0.970)  (-0.005, 0.005) |
| **HI** | 1.170 | 1.172  (0.740)  (-0.009, 0.005) | 1.180  (0.320)  (-0.021, 0.001) |
| **GTV_Dmax_ (Gy)** | 52.726 | 52.736  (0.972)  (-0.339, 0.319) | 52.736  (0.528)  (-0.845, 0.241) |
| **GTV_v95%_** | 50.246 | 50.208  (0.342)  (-0.363, 5.154) | 50.374  (0.368)  (-1.814, 18.010) |
| **Volume (cc)** | 29.700 | 34.412  (0.358)  (-10.353, 0.929) | 36.994  (0.132)  (-14.480, -0.108) |
| **PTV** | **Ground truth**  **(original CT)** | **DL-based synthetic CT** | **MRCAT** |
| **CI** | 1.074 | 1.090  (0.412)  (-0.016, 0.012) | 1.090  (0.010)  (-0.028, -0.016) |
| **DHI** | 0.900 | 0.890  (0.426)  (-0.005, 0.004) | 0.890  (0.260)  (-0.005, 0.005) |
| **HI** | 1.170 | 1.170  (0.748)  (-0.009, 0.005) | 1.170  (0.405)  (-0.021, 0.001) |
| **PTV_Dmax_ (Gy)** | 52.720 | 52.730  (0.972)  (-0.339, 0.319) | 53.020  (0.528)  (-0.845, 0.241) |
| **PTV_v95%_** | 4.500 | 4.560  (0.889)  (-0.363, 5.154) | 4.720  (0.810)  (-1.814, 18.010) |
| **Volume (cc)** | 769.000 | 769.860  (0.937)  (-10.353, 0.929) | 776.470  (0.797)  (-14.480, -0.108) |

Table S4. Dosimetric comparison of DL-based synthetic CT and MRCAT against ground truth for organs and risk (small bowel, bladder, right femur head, left femur_head, and rectum), including p-values with 95% confidence interval (CI) (below each result in DL-based synthetic CT and MRCAT synthetic CT).

| **OARs** | **Value** | **Ground truth**  **(original CT)** | **DL-based**  **synthetic CT** | **MRCAT** |
| --- | --- | --- | --- | --- |
| **Small bowel** | **D_mean_ (Gy)** | 16.900 | 18.718  (0.641)  (-6.294, 2.658) | 16.428  (0.815)  (-1.878, 2.822) |
|  | **V_5Gy_ (%)** | 60.086 | 61.780  (0.893)  (-16.365, 12.977) | 55.444  (0.550)  (-4.209, 13.493) |
|  | **V_10Gy_ (%)** | 57.802 | 60.210  (0.852)  (-17.395, 12.579) | 53.316  (0.543)  (-3.902, 12.874) |
|  | **V_20Gy_ (%)** | 39.746 | 42.261  (0.583)  (-16.982, 5.952) | 39.527  (0.964)  (-5.467, 5.905) |
|  | **V_30Gy_ (%)** | 23.228 | 27.914  (0.440) (-11.485, 2.114) | 23.779  (0.844)  (-3.805, 2.705) |
|  | **Volume (cc)** | 1901.718 | 1733.418  (0.626) (-227.841, 564.441) | 1818.154  (0.739)  (-206.701, 373.829) |
| **Bladder** | **D_mean_ (Gy)** | 31.084 | 33.366  (0.070)  (-3.435, -1.129) | 32.586  (0.169)  (-2.616, -0.388) |
|  | **V_20Gy_ (%)** | 81.434 | 91.625  (0.029)  (-13.978, -6.406) | 90.004  (0.074)  (-12.986, -4.155) |
|  | **V_30Gy_ (%)** | 48.916 | 56.473  (0.063)  (-11.233, -3.880) | 54.822  (0.193)  (-10.602, -1.211) |
|  | **Volume (cc)** | 77.322 | 132.124  (0.064)  (-81.556, -28.048) | 155.168  (0.030)  (-1073.159, -48.533) |
| **Rectum** | **D_mean_ (Gy)** | 30.670 | 31.704  (0.539)  (-2.949, 0.881) | 31.888  (0.651)  (-4.319, 1.883) |
|  | **V_5Gy_ (%)** | 93.781 | 83.146  (0.313)  (-0.812, 22.082) | 92.416  (0.616)  (-1.759, 4.489) |
|  | **V_10Gy_ (%)** | 91.015 | 89.829  (0.760)  (-3.232, 5.694) | 88.684  (0.533)  (-1.915, 6.577) |
|  | **V_20Gy_ (%)** | 82.812 | 83.678  (0.873)  (-7.195, 5.463) | 82.684  (0.976)  (-9.076, 9.556) |
|  | **V_30Gy_ (%)** | 55.928 | 60.302  (0.434)  (-10.626, 1.879) | 62.527  (0.477)  (-17.057, 3.858) |
|  | **Volume (cc)** | 66.358 | 87.454  (0.028)  (-28.895, -13.297) | 93.390  (0.084)  (-41.717, -12.347) |
| **Femur head**  **(right)** | **D_mean_ (Gy)** | 12.354 | 12.094  (0.572)  (-0.265, 0.785) | 12.190  (0.588)  (-0.182, 0.510) |
|  | **V_5Gy_ (%)** | 80.721 | 80.736  (0.994)  (-2.199, 2.170) | 81.956  (0.564)  (-3.677, 1.207) |
|  | **V_10Gy_ (%)** | 53.990 | 49.679  (0.281)  (0.013, 8.608) | 53.068  (0.706)  (-1.902, 3.746) |
|  | **V_20Gy_ (%)** | 17.444 | 18.039  (0.848)  (-2.091, 1.501) | 17.049  (0.593)  (-0.793, 2.184) |
|  | **V_30Gy_ (%)** | 3.733 | 3.823  (0.868)  (-0.726, 0.545) | 3.271  (0.475)  (-0.266, 1.189) |
|  | **Volume (cc)** | 117.790 | 108.630  (0.254)  (0.615, 17.705) | 109.848  (0.325)  (-0.856, 16.740) |
| **Femur head**  **(left)** | **D_mean_ (Gy)** | 12.626 | 11.960  (0.315)  (-0.054, 1.386) | 12.226  (0.417)  (-0.150, 0.949) |
|  | **V_5Gy_ (%)** | 84.155 | 82.964  (0.684)  (-2.188, 4.569) | 83.934  (0.930)  (-2.702, 3.144) |
|  | **V_10Gy_ (%)** | 55.376 | 48.631  (0.267)  (0.243, 13.248) | 51.214  (0.262)  (0.197, 8.127) |
|  | **V_20Gy_ (%)** | 18.211 | 16.692  (0.491)  (-0.974, 4.012) | 17.085  (0.610)  (-1.404, 3.656) |
|  | **V_30Gy_ (%)** | 3.680 | 3.118  (0.258)  (0.032, 1.902) | 3.245  (0.594)  (-0.497, 1.364) |
|  | **Volume (cc)** | 113.242 | 105.644  (0.344)  (-1.200, 16.396) | 106.658  (0.377)  (-1.660, 14.828) |
